# Characterization of Microsatellites in the *Akebia trifoliata* Genome and Their Transferability and Development of a Whole Set of Effective, Polymorphic, and Physically Mapped Simple Sequence Repeat Markers

**DOI:** 10.3389/fpls.2022.860101

**Published:** 2022-03-18

**Authors:** Shengfu Zhong, Wei Chen, Huai Yang, Jinliang Shen, Tianheng Ren, Zhi Li, Feiquan Tan, Peigao Luo

**Affiliations:** ^1^Provincial Key Laboratory for Plant Genetics and Breeding, College of Agronomy, Sichuan Agricultural University, Chengdu, China; ^2^College of Forestry, Sichuan Agricultural University, Chengdu, China

**Keywords:** *Akebia trifoliata*, microsatellite, genomic collinearity analysis, transferability, angiosperm

## Abstract

*Akebia trifoliata* is a perennial climbing woody liana plant with a high potential for commercial exploitation and theoretical research. Similarly, microsatellites (simple sequence repeats, SSRs) also have dual roles: as critical markers and as essential elements of the eukaryotic genome. To characterize the profile of SSRs and develop molecular markers, the high-quality assembled genome of *A. trifoliata* was used. Additionally, to determine the potential transferability of SSR loci, the genomes of *Amborella trichopoda*, *Oryza sativa*, *Vitis vinifera*, *Arabidopsis thaliana*, *Papaver somniferum*, and *Aquilegia coerulea* were also used. We identified 434,293 SSRs with abundant short repeats, such as 290,868 (66.98%) single-nucleotide repeats (SNRs) and 113,299 (26.09%) dinucleotide repeats (DNRs) in the *A. trifoliata* genome. 398,728 (91.81%) SSRs on 344,283 loci were physically mapped on the chromosomes, and a positive correlation (*r* = 0.98) was found between the number of SSRs and chromosomal length. Additionally, 342,916 (99.60%) potential SSR markers could be designed from the 344,283 physically mapped loci, while only 36,160 could be viewed as high-polymorphism-potential (HPP) markers, findings that were validated by PCR. Finally, SSR loci exhibited broad potential transferability, particularly DNRs such as the “AT/AT” and “AG/CT” loci, among all angiosperms, a finding that was not related to the genetic divergence distance. Practically, we developed a whole set of effective, polymorphic, and physically anchored molecular markers and found that, evolutionarily, DNRs could be responsible for microsatellite origin and protecting gene function.

## Introduction

Microsatellites or tandem simple sequence repeats (SSRs), iterations of 1–6 bp nucleotide motifs, exist widely in the genomes of prokaryotic and eukaryotic organisms (Gupta and Varshney, 2000). SSRs were initially regarded as “junk DNA” or mainly used as “neutral” genetic markers. However, recent studies have documented their crucial effects on gene activity, chromatin organization, and protein function (Deng et al., 2016), particularly for SSRs within functional genes. Currently, SSRs in genes are mainly involved in regulating biological processes because SSRs in protein coding regions may lead to the acquisition or loss of gene function (Fox et al., 2019).

Furthermore, SSRs have been widely used in population genetics, comparative analysis, DNA fingerprinting, varietal identification, genetic linkage mapping, and molecular marker-assisted breeding because of their high reproducibility, codominant inheritance, multiallelic nature, abundance, and wide genome coverage (Qi et al., 2015). SSR markers were first derived from fragmented sequences such as expressed sequence tags (ESTs) and DNA libraries over a period of time. In recent years, with an increasing number of plants being sequenced, many SSR loci and markers located in the entire genome have been identified from assembled whole-genome sequences (Biswas et al., 2020; Dharajiya et al., 2020; Jian et al., 2021; Zhu et al., 2021), and they are very useful molecular tools for many crops, particularly perennial horticultural crops, or the early exploitation of plant resources. Additionally, the increasing amount of available information concerning SSR loci also provides new insights into the evolution of plants.

*Akebia trifoliata* (Thunb.) Koidz. (2*n* = 2*x* = 32), belonging to the Lardizabalaceae family of flowering plants, is a climbing woody liana plant mainly distributed in East Asia, particularly in China, Korea, and Japan (Li et al., 2010), and has recently attracted the attention of both commercial farmers and evolutionary biologists. On the one hand, it is a multipurpose plant used in traditional medicine (Jiang et al., 2020) as an edible oil plant (Su et al., 2021) and as a fruit crop (Niu et al., 2020). On the other hand, *A. trifoliata* is a representative species of the basal eudicot lineage; thus, it also plays a crucial role in the study of the early evolution of eudicots (Liu et al., 2021). However, the shortage of molecular tools such as SSRs has severely impeded both genetic improvement for economic exploitation and progress in the evolutionary biology field. Therefore, systematic study of *A. trifoliata* microsatellites is highly significant for both practical and theoretical applications.

Although a few reports on SSRs in *A. trifoliata* are available, the studies had some shortcomings, such as a comparatively small number (Li et al., 2009, 2019; Niu et al., 2019) and a lack of physical positions (Zhang et al., 2021). In this study, we identified genome-wide SSRs in *A. trifoliata* and outlined their characteristics, which will be helpful for both genomic evolution studies and molecular breeding.

## Materials and Methods

### Genomic Data and Plant Materials

The recently published genome of *A. trifoliata* subsp. *australis* by Huang et al. (2021) was not used in the present study, primarily because the corresponding assembled genome is still unavailable. Both the genome sequence with ID PRJNA671772 of *A. trifoliata* downloaded from the National Center for Biotechnology Information (NCBI) database and corresponding annotation files (unpublished) were used to characterize SSRs. Additionally, *A. trichopoda* is a basal angiosperm (Albert et al., 2013), *Oryza sativa* is representative of monocots and agricultural importance (Kawahara et al., 2013), *Vitis vinifera* is representative of core eudicots with good trace retention of genomic changes (Jaillon et al., 2007), *Arabidopsis thaliana* is a model plant (The Arabidopsis Genome Initiative, 2000), *Papaver somniferum* is a representative basal eudicot (Guo et al., 2018), and *Aquilegia coerulea* is a species closely related to *A. trifoliata* (Filiault et al., 2018). The genome sequences of these species were downloaded from the NCBI and Phytozome databases. Finally, we randomly selected 100 genotypes (Supplementary Table 1; Supplementary Figure 1) from the germplasm collections of 3,158 accessions of *A. trifoliata* from 15 provinces—Sichuan, Chongqing, Guizhou, Yunnan, Guangxi, Fujian, Zhejiang, Jiangxi, Hunan, Hubei, Anhui, Shanxi (short name: Shan), Shanxi (short name: Jin), Gansu and Henan—and they were used to test the effectiveness and polymorphism of SSR markers. All 100 plant individuals are preserved in the Germplasm Nursery at Sichuan Agricultural University Chongzhou Research Station (30°43′N, 103°65′E; Guan et al., 2022).

### Identification of Genome-Wide SSRs

Whole-genome SSRs were detected using the microsatellite identification software MicroSatellite (MISA) and default parameters (Thiel et al., 2003). Briefly, the genome sequence data were searched for single-nucleotide, dinucleotide, trinucleotide, tetranucleotide, pentanucleotide, and hexanucleotide motifs of SSRs. The minimum repeat numbers of single nucleotides and dinucleotides were 10 and 6, respectively, while that of the other motifs were five. Two SSRs were registered as compound SSRs if the interval between them was less than 100 bp. The identification of SSRs of other model plants and closely related plant species, including *A. trichopoda*, *O. sativa*, *V. vinifera*, *A. thaliana*, *P. somniferum*, and *A. coerulea*, was also conducted using the method described above. The statistics and classification of SSR types were conducted based on the MISA output results. The correlation coefficient was calculated using the Pearson method. The phylogenetic tree of seven plants was obtained from the TimeTree database.^1^ For comparative genomic analysis of SSR loci, *A. trifoliata* SSRs with 50-bp flanking sequences were extracted to determine sequence similarity with SSRs from other plants using BLAST software. The Blastn mode was chosen to conduct the sequence alignment using the parameters “-evalue 1e-10” and “-word_size 7.” Synteny analysis of *A. trifoliata* SSRs and genes was performed using MCScanX with default parameters (Wang et al., 2012).

### Development of Genome-Wide SSR Markers

To develop the SSR markers, 150-bp sequences of the flanking regions of the SSR loci were selected to design the primer pairs. Only one pair of primers was designed for each SSR locus. Two PERL scripts, “p3_in.pl” and “p3_out.pl,” provided by the MISA package were used to convert the data format to one suitable for primer design. These modified flanking sequences were then searched, and primers were designed using Primer3 (Untergasser et al., 2012), with PCR product sizes ranging from 100 to 300 bp, primer lengths ranging from 18 to 23 bp, melting temperatures ranging from 50 to 65°C, and GC contents ranging from 40 to 60%.

### Leaf Sampling and DNA Extraction

Young leaves of the 100 selected accessions were sampled from the young branches of the parent plants, immediately frozen in liquid nitrogen, and finally stored in a freezer at −80°C for subsequent study. The genomic DNA of the sampled young leaves was extracted using a previously described CTAB protocol (Murray and Thompson, 1980). Each DNA sample was applied to examine the polymorphisms of the SSR markers.

### Validation of Developed SSR Markers

For the polymorphic marker survey and validation, 100 SSR markers with dinucleotide motifs containing a minimum of 25 repeats (≥50 bp) were randomly selected to screen for PCR amplification. PCR (25-μl volume) was performed in a PTC-200 thermocycler (MJ Research, Watertown, MA, United States). Each PCR mixture contained each SSR primer at a concentration of 200 nmol/L, 0.2 mmol/L dNTPs, 1.5 mmol/L MgCl_2_, 1 unit of Taq polymerase, and 60 ng of template DNA. PCR was performed as follows: 94°C for 1 min, followed by 30 cycles of 94°C for 45 s, 55°C–60°C for 30 s, and 72°C for 30 s and 10 min at 72°C for the final amplification. Next, 4 μl of each PCR product was mixed with 2 μl of loading buffer and loaded onto a 6% nondenaturing polyacrylamide gel for separation and visualization by capillary electrophoresis. Mapping and visualizing of the SSR markers on the *A. trifoliata* chromosome map was conducted by TBtools (Chen et al., 2020).

## Results

### Total SSRs in the *Akebia trifoliata* Genome

**Figure 1:**
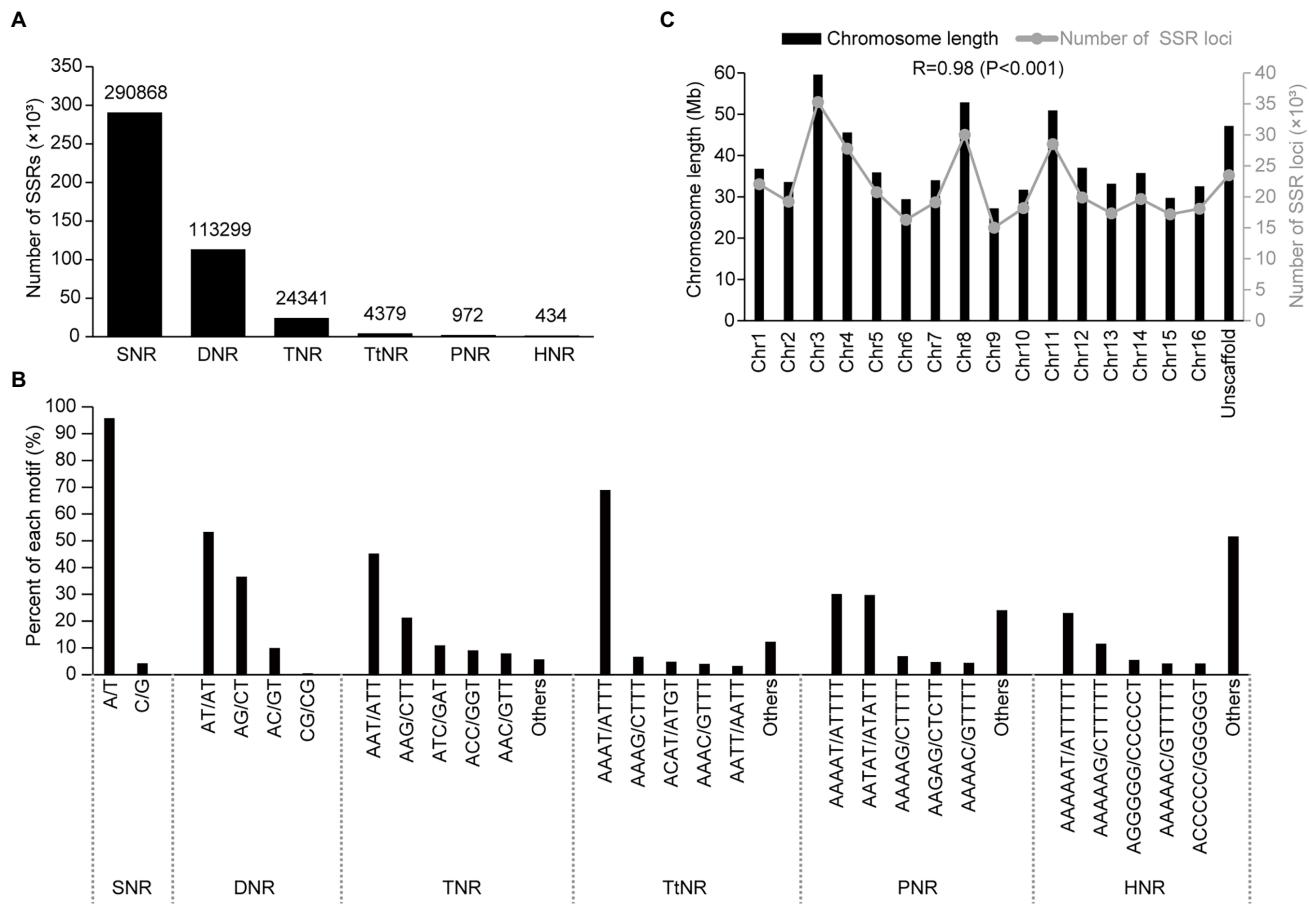
Distribution characteristics of the total simple sequence repeats (SSRs) in the *Akebia trifoliata* genome. **(A)** Number distribution of different SSR types. **(B)** Frequency distribution of different motifs within the same types. **(C)** Correlation between the chromosome length and SSR number.

A total of 434,293 SSRs with an average density of 665.28 per Mb were identified in the genome sequence (652.80 Mb) of *A. trifoliata* (Table 1). Among the identified genomic SSRs, the number of SSRs generally decreased as both the repeat unit length and repeat time increased (Figure 1A; Supplementary Table 2). For example, single-nucleotide repeats (SNRs) were the most abundant at 290,868 (66.98%), followed by dinucleotide repeats (DNRs; 113,299, 26.09%) and trinucleotide repeats (TNRs; 24,341, 5.60%). Long motifs exhibited relatively low numbers and proportions, such as tetranucleotide repeats (TtNRs), pentanucleotide repeats (PNRs), and hexanucleotides (HNRs), with values of 4,379 (1.01%), 972 (0.22%), and 434 (0.10%), respectively. Additionally, among the identified SSRs, the major types of SSR motifs were combinations of “A” and “T” repeats in the *A. trifoliata* genome, such as “A/T” in the total SNRs (95.76%), “AT/AT” in the total DNRs (53.35%), “AAT/ATT” in the TNRs (45.19%), and “AAAT/ATTT” in the TtNRs (68.94%; Figure 1B). Distribution analysis revealed that SSRs were widely distributed on every chromosome and that the number of SSRs was positively correlated (*r* = 0.98; *p* < 0.001) with the chromosomal length. The largest number of SSRs was observed on chromosome 3, while the smallest number of SSRs was observed on chromosome 9 (Figure 1C).

**Table 1:** Statistical analysis of SSRs and markers on each chromosome of *Akebia trifoliata*.

Chromosome	Chromosome length	All SSRs		SSR loci		Designed markers		HPP markers		DNR motif type of HPP markers	
		Number	Density	Number	Density	Number	Density	Number	Density	Number	Density
chr1	36,762,634	25,521	694.21	22,037	599.44	21,980	597.89	2,323	63.19	144	3.92
chr2	33,548,303	22,185	661.29	19,216	572.79	19,142	570.58	1,950	58.13	126	3.76
chr3	59,574,533	40,630	682	35,298	592.5	35,191	590.71	3,533	59.3	141	2.37
chr4	45,574,212	32,098	704.3	27,774	609.42	27,673	607.21	2,886	63.33	236	5.18
chr5	35,891,987	23,964	667.67	20,747	578.04	20,644	575.17	2,145	59.76	145	4.04
chr6	29,399,515	18,963	645.01	16,283	553.85	16,206	551.23	1,769	60.17	144	4.9
chr7	33,996,233	22,079	649.45	19,107	562.03	19,023	559.56	1,990	58.54	139	4.09
chr8	52,859,063	34,618	654.91	30,005	567.64	29,884	565.35	3,041	57.53	157	2.97
chr9	27,207,102	17,497	643.1	15,016	551.91	14,963	549.97	1,672	61.45	149	5.48
chr10	31,715,805	21,094	665.09	18,176	573.09	18,094	570.5	1,900	59.91	134	4.23
chr11	50,904,312	33,146	651.14	28,508	560.03	28,366	557.24	3,140	61.68	245	4.81
chr12	37,028,448	23,204	626.65	19,891	537.18	19,803	534.81	2,152	58.12	207	5.59
chr13	33,184,899	20,103	605.79	17,327	522.14	17,267	520.33	1,835	55.3	124	3.74
chr14	35,751,958	22,716	635.38	19,641	549.37	19,554	546.94	2,028	56.72	122	3.41
chr15	29,711,039	19,960	671.8	17,178	578.17	17,108	575.81	1,859	62.57	135	4.54
chr16	32,539,586	20,950	643.83	18,079	555.6	18,018	553.73	1,937	59.53	219	6.73
Unscaffold	47,147,008	35,565	754.34	23,486	498.14	–	–	–	–	–	–
Total	652,796,637	434,293	665.28	367,769	563.37	342,916	525.3	36,160	55.39	2,567	3.93

Two SSRs with less than 100 bp between them were registered as compound SSR loci. Density means the number of SSR loci or markers per 1 Mb chromosome length.

### Physical Mapping of SSRs on Chromosomes

**Figure 2:**
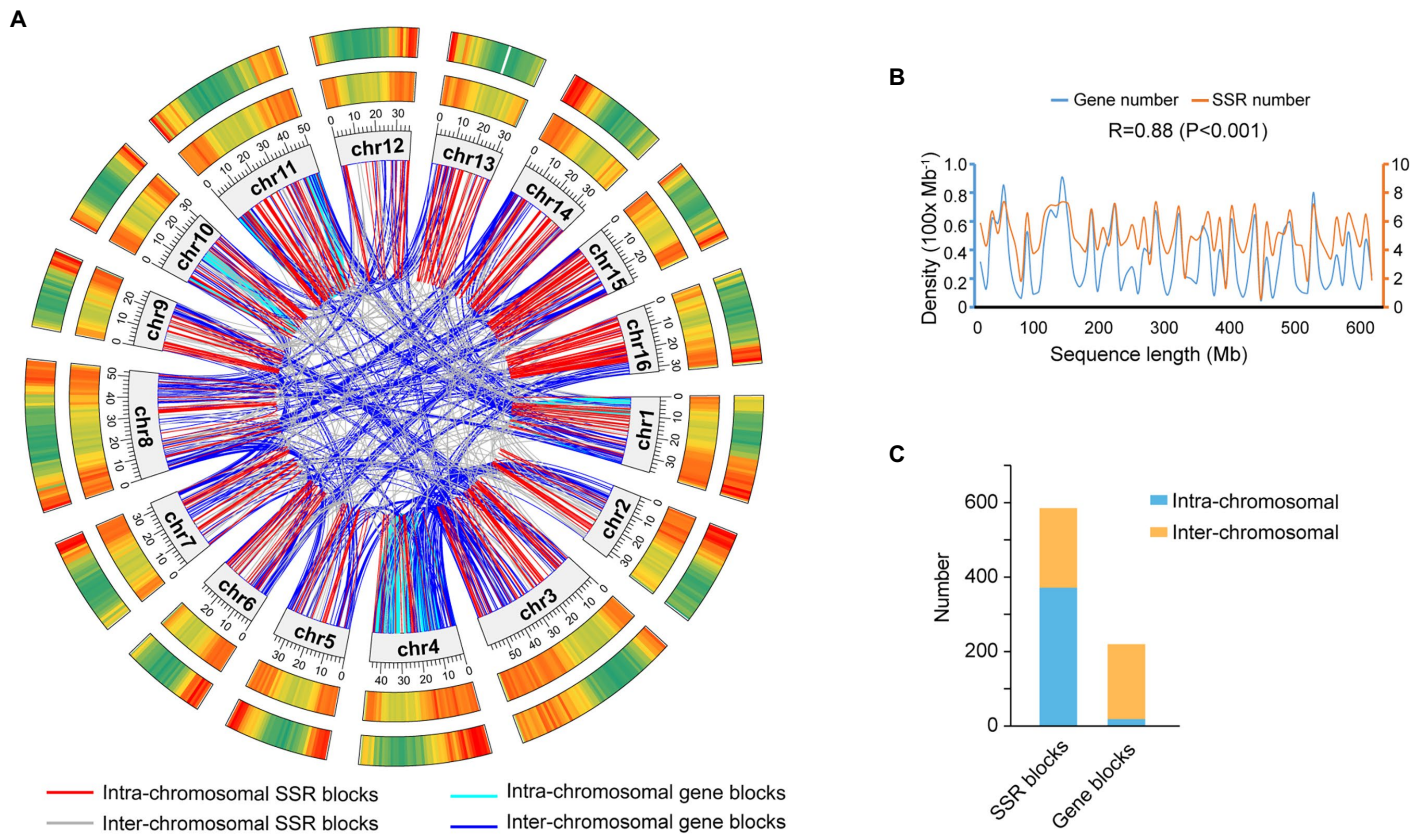
Location and collinearity analysis of SSR loci and genes on the *Akebia trifoliata* chromosomes. **(A)** Comparative physical maps of SSR loci and genes, in which the rings from the inner circle to the outer circle show the nucleotide positions on the 16 assembled chromosomes (Mb), gene density, and SSR locus density; the densities are plotted in a 1 Mb sliding window; and gradient colors from green to red in the circles represent the densities from lowest to highest, respectively. **(B)** Density correlation between gene loci and SSR loci on the 16 chromosomes. **(C)** Statistical analysis of SSRs and intrachromosomal and interchromosomal gene collinearity blocks.

Among 434,293 SSRs, 398,728 (91.81%) on 344,283 loci were physically mapped on the 16 high-quality assembled pseudochromosomes, while 35,565 (8.19%) on 23,486 loci were assigned on 47.15 Mb unassembled scaffolds (Figure 2A; Table 1). The density distribution between SSR loci and functional genes on each chromosome was highly correlated, and their Pearson correlation coefficient was 0.88 (*p* < 0.001; Figures 2A,B). The distribution of both SSR loci and genes showed that their density in the middle of each chromosome was lower, while that at the ends of each chromosome was higher. Additionally, the collinearity blockade of SSR loci was mainly intrachromosomal, while that of functional genes was interchromosomal (Figures 2A,C).

### Development of Genome-Wide SSR Markers

**Figure 3:**
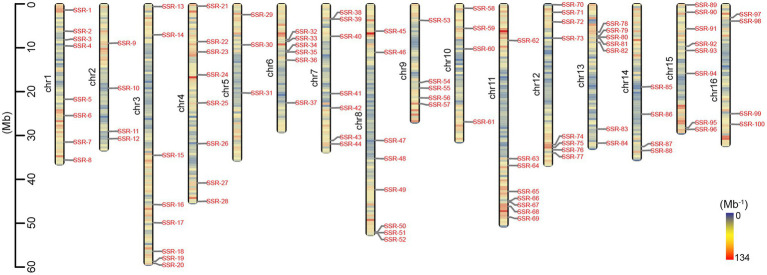
Distribution of high-polymorphism-potential (HPP) SSR markers on the physical map of *Akebia trifoliata*. The left axis displays the length of chromosomes (Mb), and the color depth on the chromosomes represents the density of HPP markers (Mb^−1^).

In total, 342,916 (99.60%) of 344,283 loci on the 16 pseudochromosomes developed potential primer pairs according to the 150-bp flanking sequences, while only 36,160 (10.54%) of 342,916 primer pairs had SSR lengths larger than 50 bp; therefore, they were generally viewed as high-polymorphism-potential (HPP) SSR markers. The details of the SSR type, motif, length, and sequence of the 36,160 HPP markers are provided in Supplementary Table 3. In total, the density of the HPP SSR markers was also lower in the middle regions and high in the end regions of each chromosome (Figure 3). The average number of HPP markers on every chromosome was 2,260, ranging from 1,672 on chromosome 9 to 3,533 on chromosome 3 (Table 1; Figure 3), and the correlation coefficient between the number of HPP SSR markers and chromosomal length was 0.99 (*p* < 0.001). However, a weak relationship (*r* = 0.35; *p* = 0.184) was found between the DNR number of HPP SSR markers and chromosomal length, and the DNR density was highest at 6.73 per Mb on chromosome 16 and lowest at 2.37 per Mb on chromosome 3. Additionally, among the 36,160 HPP SSR markers, the compound type was the most abundant (90.63%) and the DNR type was the second most abundant (7.10%; Table 1; Supplementary Table 3).

### Validation of SSR Markers

In total, 96 and 83 of the 100 randomly selected HPP SSR markers, which were designed according to the DNR type and were evenly distributed on each of 16 chromosomes (Figure 3), produced clear bands and polymorphic bands, respectively, in different genotypes of the natural population (Supplementary Figure 2). In the 83 polymorphic markers, 335 alleles were detected. The number of alleles for each polymorphic SSR ranged from 1 to 12, with a mean of 4.04 (Supplementary Table 4).

### SSR Characteristics of Several Evolutionarily Important Species

Among the seven species under investigation, the number of identified SSR loci was positively correlated (*r* = 0.79; *p* = 0.03) with genome size (Supplementary Table 5). The largest genome (*Papaver somniferum*; 2715.53 Mb) contained the most (563,800) SSRs, while the smallest genome (*Arabidopsis thaliana*; 119.67 Mb) contained the fewest (50,092) SSRs. SNRs were the most abundant motif in all species, in which “A/T” was the most abundant repeat unit, ranging from 86.28 (*O. sativa*) to 98.97% (*A. thaliana*). Additionally, “AT/AT” and “AG/CT” in DNRs were the most abundant repeat units in the other six species, similar to the results for *A. trifoliata*. By contrast, an obvious difference was found in the motif of TNRs between the monocot species *O. sativa* and the other species, and the number of “CCG/CGG” motifs was 14,217 (47.49%) in the TNRs of *O. sativa*, while the number was no more than 200 or the proportion was less than 1% in the other six species. The frequencies of TtNRs, PNRs, and HNRs were very low in the seven investigated species.

### Cross-Species Comparison of SSRs

**Figure 4:**
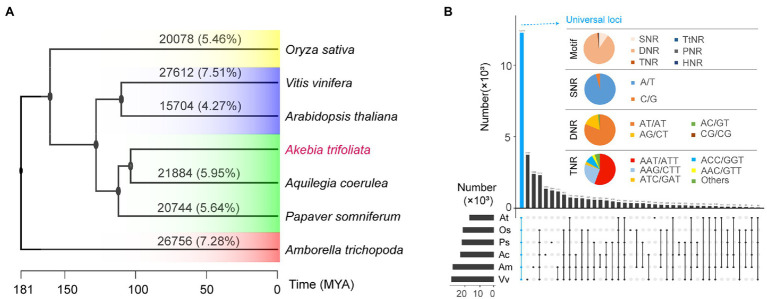
Comparative analysis of SSR loci in seven species. **(A)** Sequence similarity of SSR loci in *Akebia trifoliata* matched with the other six species; the phylogenetic tree was downloaded from the TimeTree database. **(B)** Repeat unit types of homologous SSRs among different species and motifs of the universal loci of seven species.

The number and proportion of sequence-based homologues of SSR loci identified by *in silico* comparative genome mapping showed that only a low proportion, ranging from 4.27% (*A. thaliana*) to 7.51% (*Vitis vinifera*), of SSR loci in *A. trifoliata* were homologous to those in the other six species, and most abundant SSR loci showed species-specific characteristics (Figure 4A). The relationship of homologous SSRs was not consistent with the phylogenetic relationships based on functional genes. For example, the proportion of homologous SSRs between *A. trifoliata* and the distant ancient basal angiosperm *Amborella trichopoda* was 7.28%, while that between *A. trifoliata* and the closely related species *Aquilegia coerulea* was 5.95% (Figure 4A). Although SNRs were the most abundant type among SSRs in all seven plants (Figure 4B), the homologous SSR loci were further classified by aggregates and intersections. Among universal SSR loci, the most abundant type was DNRs (88.64%), not SNRs (9.63%; Figure 4B; Supplementary Table 5). Additionally, the main types among universal homologous DNRs and TNRs were “AT/AT” (81.30%) and “AAT/ATT” (55.73%), respectively (Figure 4B).

## Discussion

### Application Prospects of the Identified Genome-Wide SSRs in *Akebia trifoliata*

As an edible and healthy fruit crop, *A. trifoliata* has high potential for commercial cultivation (Niu et al., 2020) and rapid improvement of important agronomic traits such as disease resistance (Yu et al., 2021) and fruit yield (Yang et al., 2021) by molecular marker-assisted selection. Evolutionarily, *A. trifoliata* is a classic basal eudicot species that plays a crucial role in elucidating genome events during the early stage of growth (Liu et al., 2021). In the present study, we identified 434,293 SSRs from the *A. trifoliata* genome (Figure 1A; Table 1), and 398,728 (91.81%) of 344,283 loci were physically mapped on 16 pseudochromosomes, of which 19,085 SSRs on 12,276 loci were universal among all angiosperms. These SSRs are abundant and valuable resources to develop molecular markers in applied research and elucidate microsatellite evolution in plant genomes.

### Developing a Set of Effective, Polymorphic, and Physically Mapped SSR Markers for Practical Application

In applied research, SSRs are useful markers for plant genetic improvement (Deng et al., 2016). To date, few SSR markers have been developed and successfully applied in *A. trifoliata*. Initially, only 11 SSR markers were developed from an AC-enriched genomic library (Li et al., 2009); they were subsequently applied in the genetic diversity analysis of *A. trifoliata* (Li et al., 2019). A subsequent study reported that 9,494 SSRs and 100 EST-SSR markers were identified by *de novo* sequencing of the *A. trifoliata* transcriptome (Niu et al., 2019). However, unfortunately, the uncertainty regarding the physical positions has limited their wide use. Recently, 851,957 SSRs were identified from genome survey data using the whole-genome shotgun strategy (Zhang et al., 2021), but information on their physical positions remains lacking. In *A. trifoliata* research, SSR markers have not been widely applied because of their small number (Li et al., 2009), the lack of information on physical positions (Niu et al., 2019; Zhang et al., 2021), or the overabundance of markers to effectively select (Zhang et al., 2021). In the present study, the number of identified SSRs in *A. trifoliata* was 434,293 (Figure 1A), which was less than the previously reported number, 851,957 (Zhang et al., 2021). The reason might be the drawback of genome surveys, which can produce fragmented and redundant data (Hudson, 2008).

Among 344,283 loci including 398,728 SSRs physically mapped on 16 high-quality assembled pseudochromosomes, 342,916 (99.60%) could be used to design potential primer pairs according to the characteristics of the 150-bp flanking sequences, such as the GC content, specificity and DNA segmental structure, and 36,160 of these could be HPP SSR markers because they had a repeat length of at least 50 bp (Supplementary Table 3). Although some differences were found in the SSR density among different chromosomes (Figure 2A; Supplementary Table 3), the number of HPP SSRs on every chromosome was more than 1,600, meeting the requirements for SSR markers as molecular tools, such as in population density determination, molecular marker-assisted selection, and genetic mapping. Therefore, that the HPP SSRs in *A. trifoliata* were present genome wide is reasonable. Additionally, the number (36,160) of HPP SSRs was only 10.50% of the total (344,283) physically anchored SSR loci, effectively preventing the number of markers from being too high to effectively select for practical applications and therefore enhancing predictability. At the same time, that 96 and 83 of the 100 randomly selected HPP SSR markers produced clear bands and polymorphic bands, respectively, in the natural population, including 100 genotypes (Supplementary Figure 2), also further supported that the developed HPP SSRs in the study were highly reliable and polymorphic. Importantly, clear knowledge of the physical positions of both HPP SSR markers (Supplementary Table 3) would accelerate the application of the markers in theoretical and practical research.

In the past, SSRs were not usually affected by selective pressure and therefore exhibited evolutionarily neutral characteristics, which are critical traits of widely used markers during crop gene mapping (Luo et al., 2005). However, some evidence has shown that the TNR type, particularly in coding regions, could be evolutionarily selective or functionally adaptive (Moxon and Wills, 1999; Everett and Wood, 2004). Comparative studies of both coding and noncoding regions in different species have confirmed that only tri- and hexanucleotides are present in excessive numbers with a large range of repeat unit sizes compared with other types (Tóth et al., 2000). Of the 36,160 HPP SSRs, only 18 (0.05%) were TNRs and HNRs (Supplementary Table 3), suggesting that their dominant selective character was still neutral. Obviously, the 36,160 HPP SSR markers identified and physically mapped on chromosomes were highly effective and valuable polymorphic marker tools for various research projects.

### SSR Characteristics Suggest That *Akebia trifoliata* Is More Similar to Grass Than to Trees

Previous studies have shown that the SSRs of plants have common characteristics, such as enrichment at chromosomal ends (Lu et al., 2019), a larger proportion of “A/T” compared with “G/C” in SNRs and, a larger proportion of “AN/NT” compared with “GN/NC” in DNRs (The *Arabidopsis* Genome Initiative, 2000; Jaillon et al., 2007; Kawahara et al., 2013; Lu et al., 2019). These characteristics could be explained by fewer genes being present in centromere regions (Oko et al., 2020), the high association between SSRs and nonrepetitive DNA (Morgante et al., 2002), and the abundant poly(A) tail structures in downstream genes (Buschiazzo and Gemmell, 2006). In the present study, many SSR loci, such as HPP SSRs, were mainly enriched in chromosomal end regions except on chromosomes 2 and 13 (Figure 2B), and the number of both “AT/AT” and “AG/CT” was greater than that of both “AC/GT” and “CG/CG” in *A. trifoliata* as well as in the other six species (Supplementary Table 5), suggesting that the SSRs in *A. trifoliata* have many common characteristics of plant genomic SSRs. Various studies have reported that among grasses, such as *Brachypodium distachyon*, *Sorghum bicolor*, *O. sativa*, *A. thaliana*, and *Medicago truncatula*, short motifs of SSRs, including SNRs, DNRs, and TNRs, are abundant compared with long motifs (Sonah et al., 2011; Kawahara et al., 2013). By contrast, the number of long motifs of SSRs, particularly HNRs, was far larger than that of short motifs in many tree species, such as *Prunus persica*, *Salix babylonica*, *Jatropha curcas*, and *Morus notabilis* (Xia et al., 2017). *Akebia trifoliata* is a perennial woody liana plant; similar to platypus in the animal kingdom, it has some mixed features of both grass and trees. The number (428,508, 98.67%) of SSRs with short motifs (from 1 to 3 repeat units) was far greater than that (5,785, 1.33%) of SSRs with long motifs (from 4 to 6) in our study (Figure 1A; Supplementary Table 5). Therefore, concerning the SSR unit type, *A. trifoliata* was more similar to grass than to trees.

### Transferability of SSR Loci Among Angiosperms

Transferability can be a critical factor influencing the use of SSRs among different species and affords some useful information concerning microsatellite evolution. In plants, some conserved SSR loci are found across cultivars, subspecies, and related species (Métais et al., 2002). For example, the primers originally developed for *Eucalyptus* spp. could be used for *Eugenia dysenterica* (Zucchi et al., 2003). Sequence homology of microsatellite markers ranging from 6.4 to 16.9% has been observed in various species, including millet, sorghum, maize, and rice (Pandey et al., 2013), and a negative correlation exists between the homology of SSRs and genetic divergence distance of species (Lin et al., 2016). A total of 19,085 homologous SSRs on 12,276 loci among seven species were representatives of all important clades of whole angiosperms (Figure 4A); additionally, no relationship was found between the number of homologous SSRs and genetic divergence distance of species (Figure 4A). Interestingly, of the 19,085 universal SSRs, the number of DNRs was 16,916 (88.64%), while that of SNRs was only 1837 (9.63%; Figure 4B). By contrast, SNRs were the most abundant (almost more than 50%) type in all seven plants, while DNRs were the second most abundant type (and third in *O. sativa*). The results suggested that the transferability of SSRs was high among all angiosperms, and the potential transferability at the genome level was not related to genetic divergence distance, further indicating that DNRs could be the most conserved type. Furthermore, the lower number (2,567; 7.10%) of DNRs among the 36,160 HPP SSRs in the *A. trifoliata* genome (Table 1) also indirectly reinforced the view that DNRs are the most highly conserved type. Therefore, they are also called protomicrosatellites and may be related to the origin of SSRs (Rose and Falush, 1998).

### DNRs Play a Critical Role in Microsatellite Origin

The most common process underlying the origination of new microsatellites is replication slip and unequal crossover during recombination, although the detailed molecular mechanism of microsatellite origination is unclear (Bhargava and Fuentes, 2010). Several important studies have agreed that DNRs play a key role during the origin of new SSRs. First, DNRs are highly enriched in chromosomal recombination hot spots and can also act as recombination hot spots (Bailey et al., 1998). Second, a significant positive linear relationship was found between the microsatellite density and level of single-copy DNA (Morgante et al., 2002), indicating that the functional genes of eukaryotic organisms could be packaged by high-density SSRs. This view was confirmed by the abundant microsatellite distribution in two flanking regions, particularly the 5′-flanking regions of genes, and DNRs were the main contributors to the increase in SSR density (Zhang et al., 2004). Third, the different proportions of the DNR type compared with other types, particularly TNRs, between the flanking regions and coding regions of genes indicated the difference in evolutionary adaptation between them (Zhang et al., 2004).

In the present study, the high correlation coefficient (*r* = 0.88) of the density between SSR loci and functional genes (Figure 2B) indicated that both SSR loci and genes originated simultaneously, but their evolutionary styles were different. Regarding the chromosomal distribution of the collinearity block, the functional genes were mainly produced by whole-genome duplication, while SSR loci were putatively produced by interchromosomal segmental duplication. Among the 19,085 universally homologous SSRs, the proportion of DNRs (88.64%; Figure 4B) was much higher than that in every species (ranging from 11.18 to 27.60%; Supplementary Table 5), while that (1.37%) of TNRs (Figure 4B) was lower than that (ranging from 5.60 to 14.46%) in every species (Supplementary Table 5). The reason could be that TNRs are biologically functionally important, while DNRs could be structurally important because DNRs, particularly in flanking gene regions, could protect the function of genes and prevent the loss of gene function during chromosomal recombination. The large difference in the proportion of TNRs among different species and high proportion (47.49%) of “CCG/CGG” in *O. sativa* (Supplementary Table 5), the sole monocot among the seven species, also indicated that TNRs, particularly in coding regions, could experience fast functional evolution, while DNRs could be structurally highly conserved. Additionally, the conservation of DNRs could widely result in larger genetic divergence of species than conserved functional genes.

Comprehensively, we physically mapped 344,283 loci carrying 398,728 of the 434,293 SSRs identified in the *A. trifoliata* genome on 16 high-quality assembled pseudochromosomes. In total, 36,160 of the 342,916 (99.60%) potential markers could be viewed as HPP SSR markers, and both their identity and polymorphism were confirmed by PCR amplification, showing that they formed a whole set of effective polymorphic SSR markers with clear chromosomal positions. Further comparative analysis of SSR characteristics suggested that *A. trifoliata* might be a grass rather than a tree. Additionally, the SSR loci of *A. trifoliata* had high potential transferability among whole angiosperms, a finding that was not related to genetic divergence distance between species, and DNRs compared with other repeat types were highly conserved. Therefore, we inferred that DNRs could play a crucial role in microsatellite origin and could be a recombination hot spot to further evolutionarily protect the function of genes by preventing functional loss due to chromosomal recombination. In conclusion, the new data and markers provide an essential genomic resource for theoretical and applied research in *A. trifoliata*.

## Data Availability Statement

The original contributions presented in the study are included in the article/Supplementary Material; further inquiries can be directed to the corresponding author/s.

## Author Contributions

SZ and WC contributed equally to the work and wrote the manuscript. SZ, PL, TR, and ZL conceived and designed this research project. SZ, HY, and JS performed the bioinformatic analysis. WC and HY performed the verification experiment using the SSR markers. PL and FT jointly supervised this work. All the authors contributed to the revisions and comments concerning the manuscript. All authors contributed to the article and approved the submitted version.

## Funding

This work was supported by grants 2019YFN0032, 2019YFS0020, 2020YJ0331, 2020JDRC0087, and 2020JDRC0086 from the Sichuan Science and Technology Program. This work was also supported by the National Natural Science Foundation of China (32101687).

## Conflict of Interest

The authors declare that the research was conducted in the absence of any commercial or financial relationships that could be construed as a potential conflict of interest.

## Publisher’s Note

All claims expressed in this article are solely those of the authors and do not necessarily represent those of their affiliated organizations, or those of the publisher, the editors and the reviewers. Any product that may be evaluated in this article, or claim that may be made by its manufacturer, is not guaranteed or endorsed by the publisher.

## Footnotes

^1^ www.timetree.org
