# Supplementary material for: Characterization of Microsatellites in the Akebia trifoliata Genome and Their Transferability and Development of a Whole Set of Effective, Polymorphic, and Physically Mapped Simple Sequence Repeat Markers
Source: Front Plant Sci. 2022 Mar 18;13:860101. doi: 10.3389/fpls.2022.860101 (PMC8971770; doi:10.3389/fpls.2022.860101)
Supplement: Supplementary file 1 [file Data_Sheet_1.ZIP › Supplementary_Figures.docx]

**Supplementary Figures**


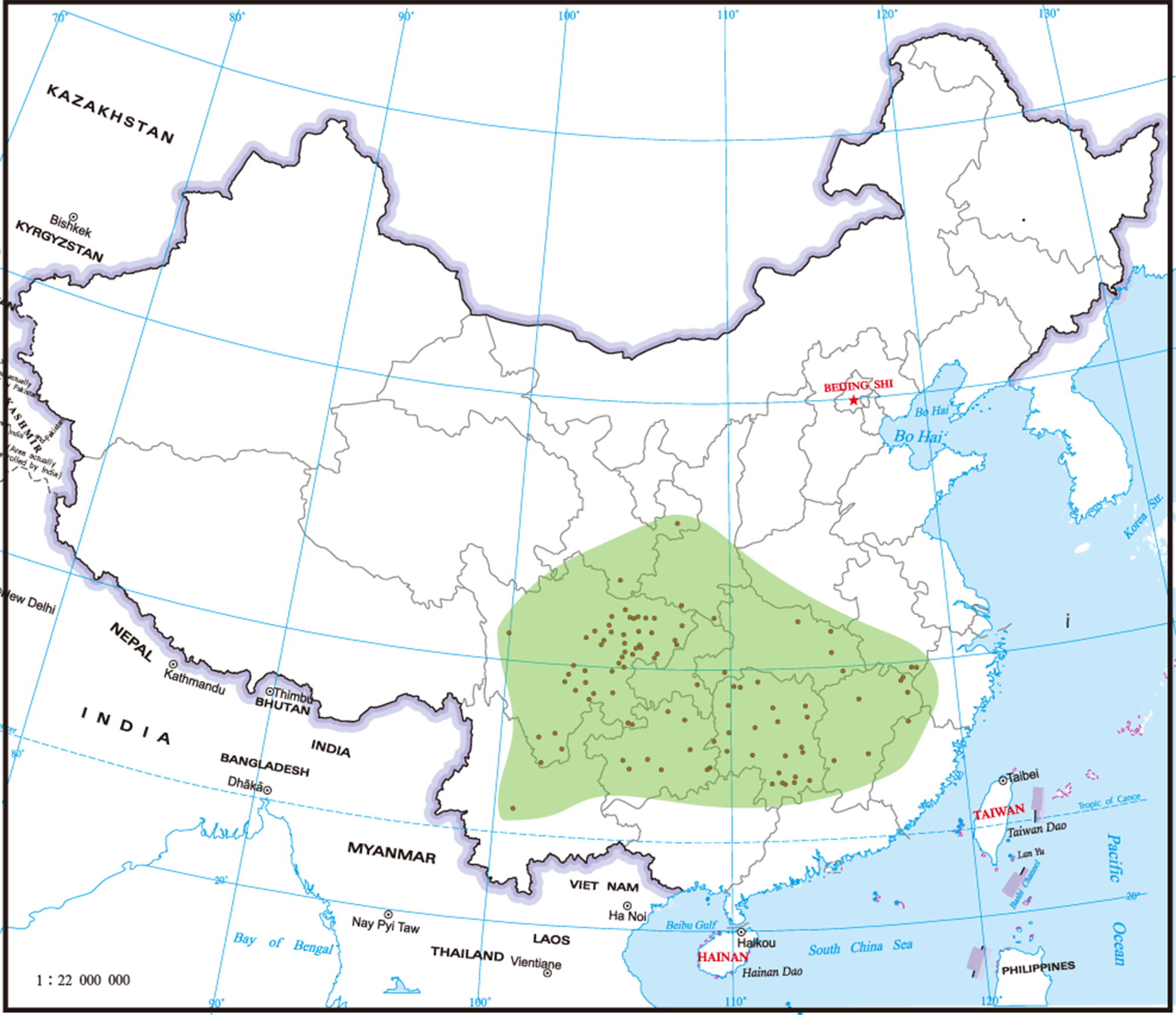


**Supplementary Figure 1.** Geographic distribution of 100 *A. trifoliata* germplasms. Each red icon represents one germplasm sample.

**
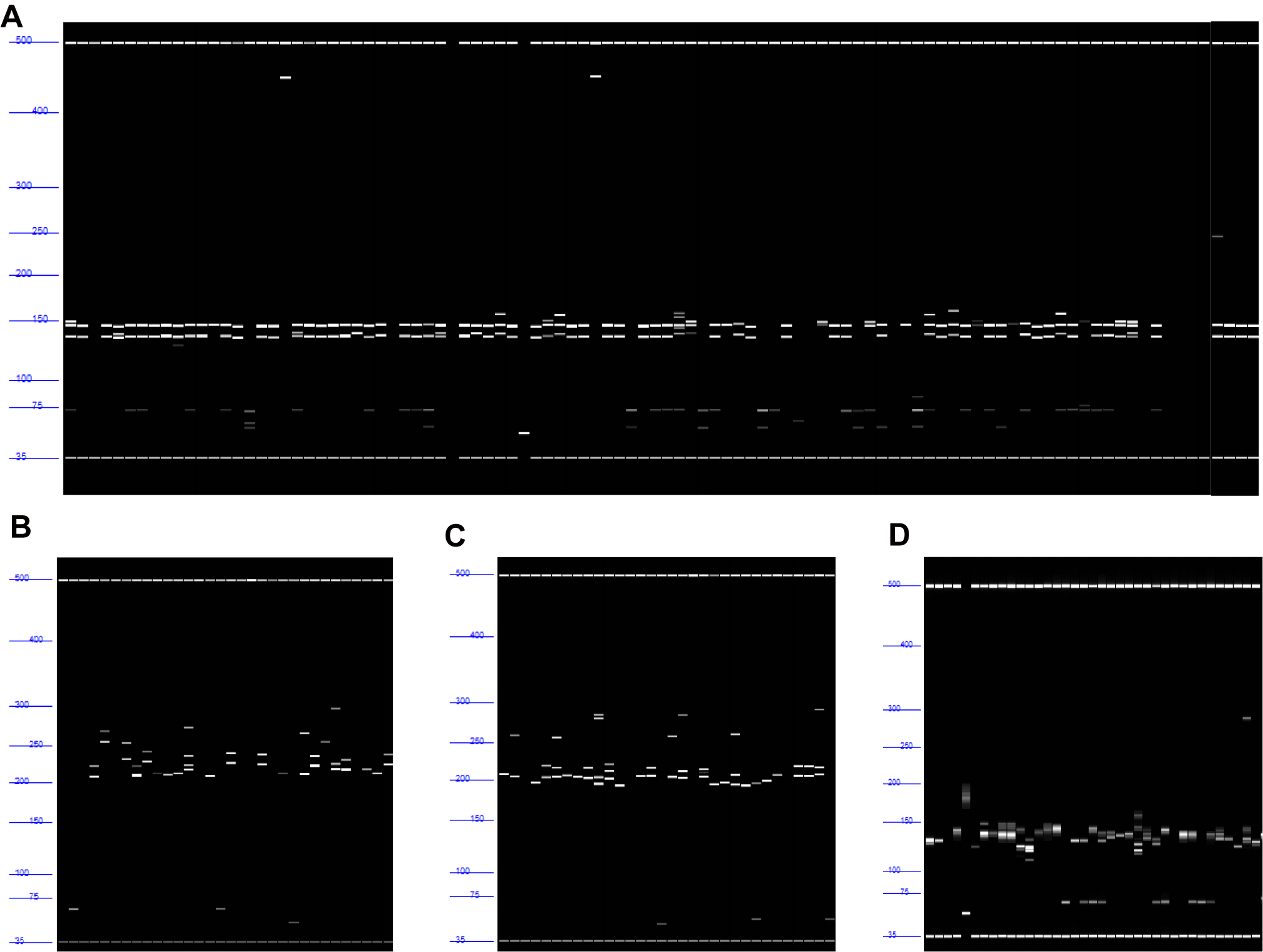
**

**Supplementary Figure 2.** Capillary electrophoresis amplification of SSR markers*.* The blue marks represent the standard molecular weight (bp). (A) An example of 100 sample amplification results of the SSR-62 marker. (B-D) Some amplification results of polymorphic markers SSR-54, SSR-64, and SSR-70, respectively.
